# Progression of the PI3K/Akt signaling pathway in chronic obstructive pulmonary disease

**DOI:** 10.3389/fphar.2023.1238782

**Published:** 2023-09-20

**Authors:** Yanhui Liu, Haobo Kong, Heping Cai, Guanru Chen, Huiying Chen, Wenyi Ruan

**Affiliations:** ^1^ Department of Clinical Pharmacy, Anhui Provincial Children’s Hospital, Hefei, Anhui, China; ^2^ Department of Respiratory Intensive Care Unit, Anhui Chest Hospital, Hefei, Anhui, China

**Keywords:** chronic obstructive pulmonary disease, PI3K/Akt signalling pathway, inhibitor, inflammation, oxidative stress

## Abstract

Chronic Obstructive Pulmonary Disease (COPD) is a chronic respiratory disease characterized by a slow progression and caused by the inhalation of harmful particulate matter. Cigarette smoke and air pollutants are the primary contributing factors. Currently, the pathogenesis of COPD remains incompletely understood. The PI3K/Akt signaling pathway has recently emerged as a critical regulator of inflammation and oxidative stress response in COPD, playing a pivotal role in the disease’s progression and treatment. This paper reviews the association between the PI3K/Akt pathway and COPD, examines effective PI3K/Akt inhibitors and novel anti-COPD agents, aiming to identify new therapeutic targets for clinical intervention in this disease.

## 1 Introduction

COPD is a heterogeneous lung disease characterized by a variety of chronic respiratory symptoms, such as difficulty breathing, coughing, sputum production, and acute exacerbation. These symptoms arise from abnormal airways (bronchitis) and/or alveolar abnormalities (emphysema), resulting in persistent and frequently progressive airflow obstruction (Venkatesan, 2023). COPD ranks as the third leading cause of death globally, following ischemic heart disease and stroke (Obeidat et al., 2018). COPD arises from the complex interplay of multiple factors. Smoking stands as a significant risk factor for COPD, while environmental exposure and genetic variation can contribute to the development or exacerbation of the disease (Lareau et al., 2019). Presently, COPD is treated with a combination of medication and non-medication strategies, smoking cessation is the primary treatment, bronchodilators and glucocorticoids are the most commonly used drugs (Sandelowsky et al., 2021). Nevertheless, COPD commonly exhibits a progressive nature, and the conventional medications used to manage it entail notable side effects. These challenges emphasize the urgent need for exploring alternative treatment modalities for COPD.

The phosphatidylinositol 3-kinase (PI3K)/protein kinase B (PKB/Akt) signaling pathway is a critical cellular pathway that regulates multiple functions such as cell survival, growth, proliferation, metastasis, and metabolism (Engelman et al., 2006; Abeyrathna and Su, 2015). The abnormal activation of the PI3K/AKT pathway is associated with various human cancers (Noorolyai et al., 2019; Glaviano et al., 2023). Additionally, it is also involved in many chronic diseases such as diabetes (Savova et al., 2023), cardiovascular diseases (Qin et al., 2021), neurological disorders (Wang Q. et al., 2022), autoimmune diseases (Cheng et al., 2022), inflammatory diseases (Xu et al., 2022), and liver diseases (Ye et al., 2023) et al. The PI3K/AKT pathway plays a crucial role in the onset and progression of numerous diseases. Recently, studies have demonstrated that inhibiting the PI3K/Akt signaling pathway reduces inflammation, apoptosis, and oxidative stress in cells, thereby playing a crucial role in COPD treatment (Sun et al., 2019).

This review comprehensively examines the structure and transduction of the PI3K/Akt signaling pathway. Additionally, it highlights the crucial role of PI3K/Akt signaling in COPD and presents a summary of potential drugs that target this pathway. The aim is to expand the therapeutic possibilities for COPD and offer innovative and effective targets for clinical intervention.

## 2 The PI3K/Akt signaling pathway

The PI3K/Akt signaling pathway plays a crucial role in various cellular regulatory processes such as cell growth, proliferation, migration, metabolism, and secretion. Furthermore, dysregulation of the PI3K/Akt signaling pathway is implicated in a diverse spectrum of human diseases, including cancer (Noorolyai et al., 2019), neurodegenerative disease (Rai et al., 2019), diabetes (Huang et al., 2018), and osteoarthritis (Sun et al., 2020).

### 2.1 The composition of PI3K

PI3Ks are a class of evolutionarily conserved intracellular lipid kinases called intracellular lipid kinases, classified into classes I, II, and III based on substrate specificity and sequence homology (Cantley, 2002). Type I PI3K can be divided into two subfamilies based on its coupling receptors. Type IA PI3K is a heterogeneous dimer composed of a p85-regulated subgroup and a p110 catalytic subgroup. It is activated by the growth factor receptor tyrosine kinase (RTK) (Katso et al., 2001). Type IB PI3K is a heterogeneous dimer composed of p101 regulatory subunits and p110-γ catalytic subunits. It is activated by G-protein-coupled receptors (GPCRs) (Voigt et al., 2006). Classes II and III PI3K are monomer types. Class II PI3K consists of a catalytic subunit similar to p110, while class III PI3K consists of a single member, Vps34 (Jean and Kiger, 2014). It is generally accepted that class I PI3K is the most widely studied, and class II and III PI3Ks have less knowledge about their specific functions. Here, we will focus on the role of Type I PI3K in COPD.

### 2.2 The composition of Akt

Akt, a serine/threonine kinase also known as PKB, belongs to the AGC protein kinase family. Three subtypes of Akt exist: Akt1 (PKBa), Akt2 (PKBb), and Akt3 (PKBc) (Risso et al., 2015). The three subtypes of Akt, closely related in mammals, possess three conserved domains: an amino terminal pleckstrin homology (PH) domain, a central kinase domain (excitation domain) highly similar to other AGC protein kinases like PKA and PKC (Peterson and Schreiber, 1999), and a carboxyl terminal regulatory domain that includes HM phosphorylation sites. The three Akt isomers exhibit high sequence similarity and structural resemblance, with Akt1 and Akt2 showing broader expression in mammals (He et al., 2021).

### 2.3 Mechanisms of PI3K/Akt pathway activation

Common mechanisms of PI3K activation involve the activation of receptor tyrosine kinase under physiological conditions. This leads to the phosphorylation of tyrosine residues and their subsequent binding to one or both SH2 domains of the PI3K splice subunit, resulting in allosteric activation of the PI3K catalytic subunit (Fruman et al., 2017). Additionally, activation of GPCR leads to allosteric activation of PI3K (Rathinaswamy et al., 2021). PI3K activation causes the transformation of PIP2 into PIP3 inside the plasma membrane, which binds specifically to the pleckstrin homlogy (PH) domain of two proteins, PDK-1 and Akt/PKB, to mediate PI3K signaling (Carnero et al., 2008).

The PI3K-dependent activation mechanism of Akt involves the interaction of Akt with PIP3, leading to its translocation to the medial membrane where its Thr308 and Ser473 sites become exposed. PDK1 phosphorylates Akt’s Thr308 site, serving as the initial step in Akt activation. Subsequently, PDK2 phosphorylates the Ser473 sites, located at the hydrophobic carboxyl group’s end, to achieve maximal Akt activation (Cole et al., 2019). Once fully activated, Akt phosphorylates downstream target proteins, thereby regulating various cellular functions such as angiogenesis, metabolism, growth, proliferation, protein synthesis, transcription, and apoptosis (Hemmings and Restuccia, 2015).

### 2.4 Regulation of the PI3K/Akt pathway

**FIGURE 1:**
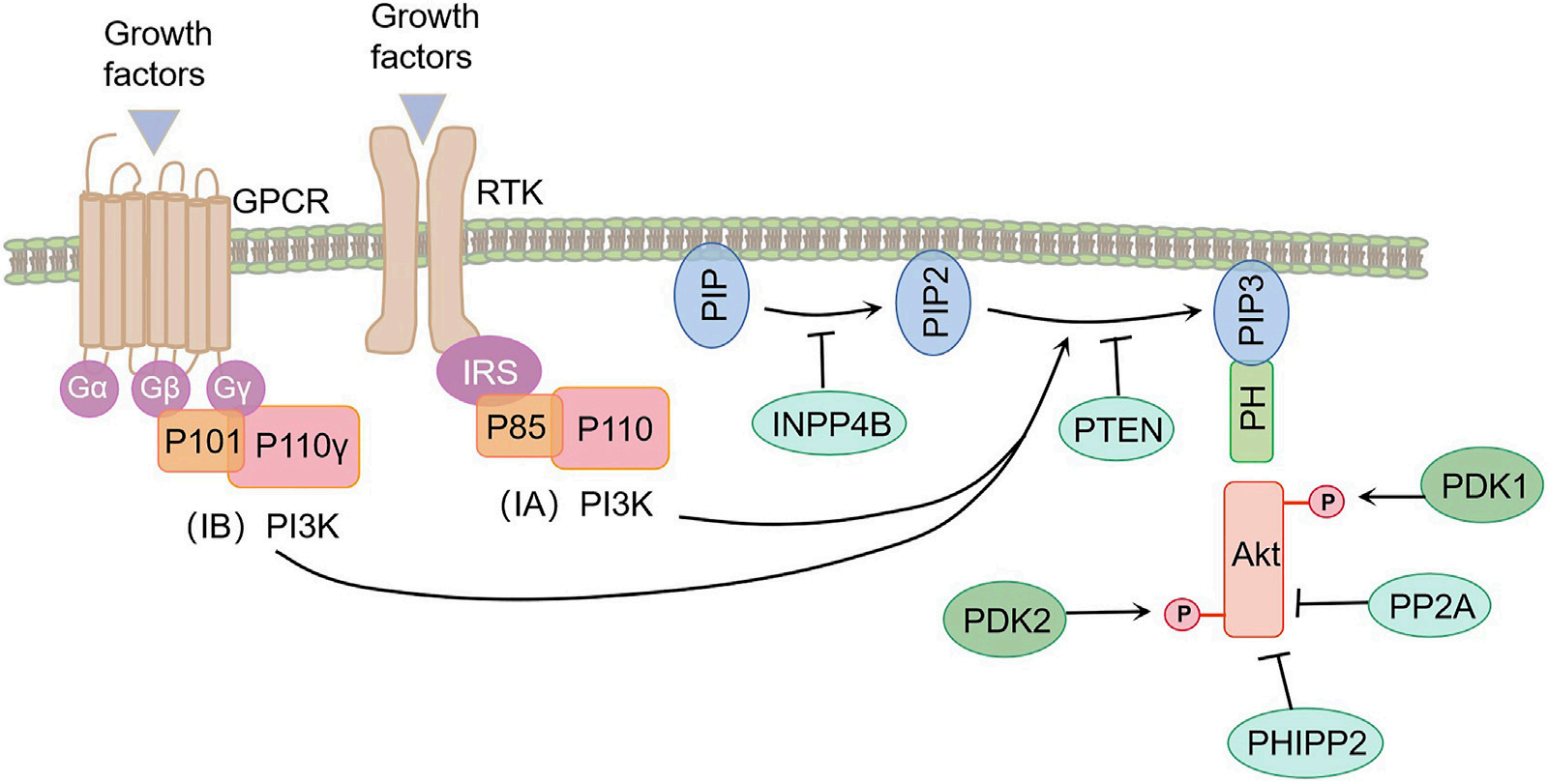
Transduction and regulatory pathways of PI3K/Akt pathway. Activation of growth factor receptor tyrosine kinase (RTK) and G-protein-coupled receptors (GPCRs) led to the activation of the PI3K catalytic subunit. Activated PI3K promotes the conversion of PIP2 to PIP3 in the medial membrane, a function that can be reversed by phosphatase and tensin homologues (PTEN). PIP3 activates Akt signaling by specifically binding to the pleckstrin homlogy (PH) domain of both PDK-1 and Akt proteins. Protein phosphatase 2 (PP2A) and the PH domain are rich in leucine repeats of the protein phosphatase (PHLPP1/2), negatively regulating the PI3K/Akt pathway.

The PI3K/Akt signaling pathway is regulated by various factors, particularly a group of phosphatases that exert negative regulatory effects. These phosphatases include phosphatase and tensin homologues (PTEN), protein phosphatase 2 (PP2A), and protein phosphatase in the PH domain rich in repeated sequences of leucine (PHLPP1/2). PTEN plays a crucial role as an upstream component of the PI3K/Akt signaling pathway. It catalyzes the specific dephosphorylation of PIP3 to produce PIP2, thereby exerting a negative regulatory effect on Akt activation (Viennet et al., 2023). Additionally, Inositol polyphosphate 4-phosphatase type II (INPP4B) inhibits Akt signaling by dephosphorylating PIP2 into PIP (Gewinner et al., 2009). PP2A, a trimeric protein, combats Akt signaling by selectively inhibiting phosphorylation of Akt’s Thr308 site (Zhang Y. et al., 2022) and dephosphorization of Akt’s Ser473 site (Hwang et al., 2013). PHLPP1/2 primarily dephosphorylates Akt at Ser473 sites. Activation of the PI3K pathway leads to stable levels of PHLPP1 and a surge of PHLPP2, both of which attenuate Akt signaling (Chen et al., 2011). Therefore, in the absence of PTEN, PHLPP2 replaces its role in attenuating the output of the PI3K/Akt pathway (Figure 1) (Chen et al., 2014).

## 3 The PI3K/Akt signaling pathway and its role in the pathogenesis of COPD

### 3.1 The interplay between inflammation and oxidative stress in the pathogenesis of COPD

Increasing evidence suggests that inflammation and oxidative stress are interconnected pathophysiological processes (Biswas, 2016). In COPD, activated inflammatory cells in the lungs release numerous inflammatory factors that trigger the production of oxygen free radicals, leading to oxidative stress (Guo et al., 2022). Moreover, oxidative stress can amplify lung inflammation by activating multiple signaling pathways within the cells (Barnes, 2022). This closely intertwined process frequently coexists across various chronic diseases, in addition to COPD (Neves et al., 2021), there are inflammatory bowel disease (Tian et al., 2017), alcoholic liver disease (Yang et al., 2022), diabetes (Grabež et al., 2022), neuroinflammatory disease (Xue et al., 2019) and other chronic inflammatory diseases.

### 3.2 The PI3K/Akt pathway and inflammation in COPD

**FIGURE 2:**
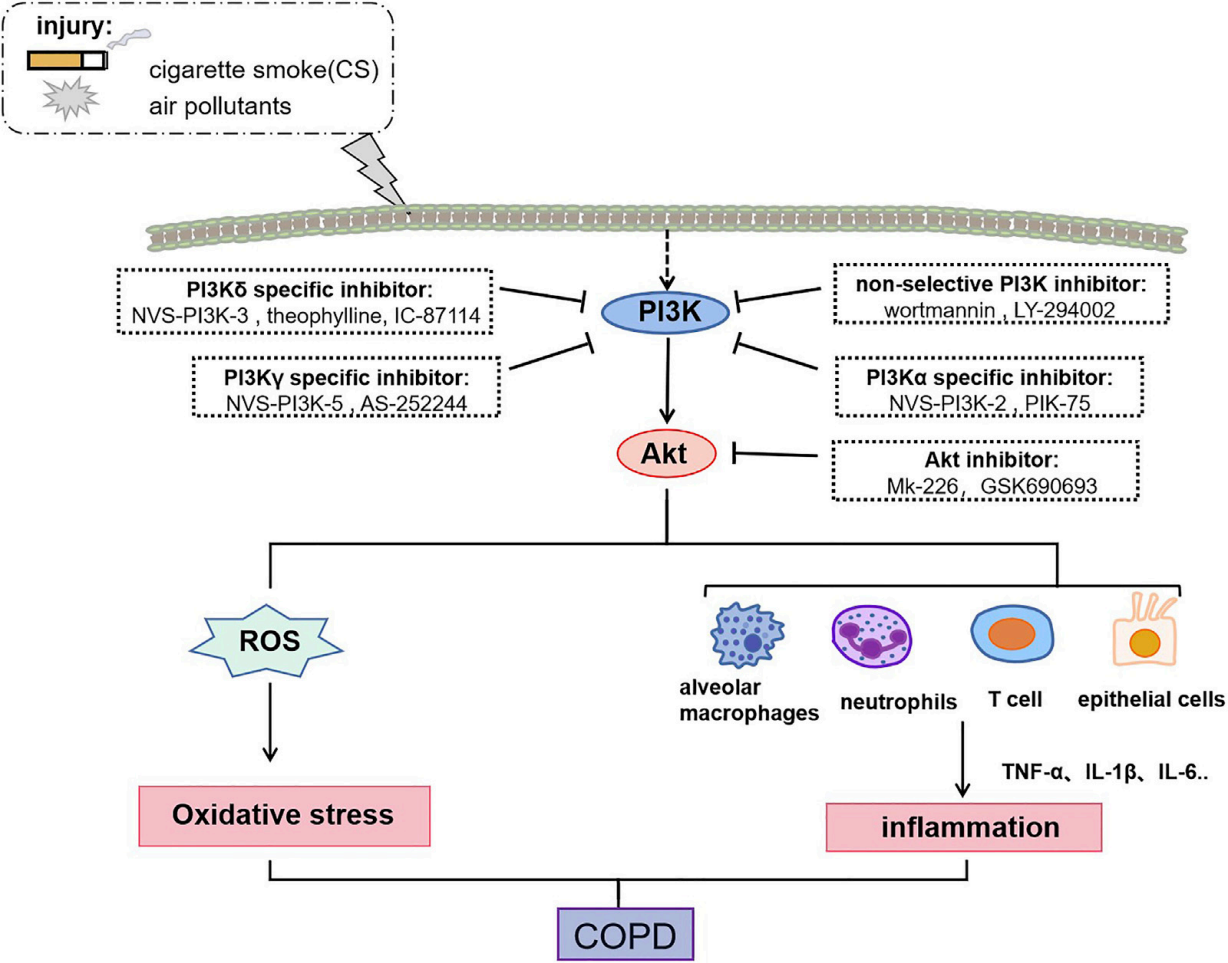
Role of PI3K /Akt in COPD regulation. The activation of PI3K/Akt is involved in COPD formation and can exacerbate COPD progression through inflammatory and oxidative stress. Specifically, activated PI3K/Akt promotes the release of ROS, as well as promotes the secretion of multiple pro-inflammatory cytokines by alveolar macrophages, neutrophils, T-lymphocytes, and epithelial cells to participate in COPD pathogenesis. Moreover, small molecule inhibitors targeting the PI3K/Akt signaling pathway can treat COPD through inhibiting oxidative stress and inflammation.

COPD is a progressive inflammatory lung condition caused by the inhalation of cigarette smoke and other toxic external particulate matter, such as air pollution and biomass fuels. Chronic inflammation of the small airways, known as bronchiolitis, serves as the primary catalyst for COPD development (Brightling and Greening, 2019). Various cytokines secreted by alveolar macrophages, neutrophils, T lymphocytes, B lymphocytes, and structural cells like epithelial, endothelial, and fibroblasts contribute to this inflammatory response (Barnes, 2016). Recent research indicates that smoking acts as an initial trigger for activating innate immune system cells, with tobacco smoke stimulating the PI3K/Akt pathway and exacerbating the inflammatory response in monocytes. Furthermore, elevated PI3K signaling has been linked to sustained inflammation in individuals with COPD, just as shown in Figure 2 (Lee et al., 2018). Furthermore, inflammation underlies significant complications in COPD, including heart and lung diseases, respiratory failure, and cancer (Byrne et al., 2015).

Zhang et al. (2017) discovered that PI3K signaling was activated in alveolar macrophages of COPD mice, leading to a significant increase in pro-inflammatory cytokines such as TNF-α, IL-1β, and IL-6, thereby enhancing inflammatory responses. Subsequent studies demonstrated that activation of the PI3K/Akt signaling pathway promoted polarization of macrophages in COPD from M1 to M2 phenotypes. The ratio of M1 to M2 macrophages following monocyte polarization has been linked to various inflammatory diseases (Zhang et al., 2023), and an elevated ratio of M2 macrophages has been implicated in lung inflammation (Lu et al., 2017). Neutrophil infiltration in the submucous membrane of the airway is the main driver of airway inflammation in COPD and is regulated by helper T cells 17 (Th17) and macrophages. Macrophages secrete inflammatory mediators that act as chemical inducers, increasing neutrophil infiltration in the airways and promoting lung injury and inflammatory responses in COPD patients (Holl et al., 2013). It is now evident that inflamed airways are exposed to hypoxia, triggering neutrophil degranulation and enhancing their potential for tissue damage. Hoenderdos et al. (2016) discovered that inhibiting the PI3K signaling pathway contributes to the suppression of neutrophil degranulation, suggesting that PI3K plays a crucial role in this process. Additionally, inhibiting Akt phosphorylation had no impact on degranulation regulation, implying that heightened neutrophil reactivity is a result of early PI3K/Akt signaling. Yanagisawa et al. (2017) demonstrated that PI3K signaling is more active in bronchial epithelial cells of COPD patients. In contrast, the negative regulator of PI3K, PTEN, is frequently mutated or absent in the airway epithelial cells of smokers. Knockdown of PTEN leads to significant Akt phosphorylation and increased secretion of pro-inflammatory cytokines (e.g., IL-6, IL-B-induced CXCL8, etc.). Hence, activating PTEN could be an effective approach to impede the progression of COPD.

### 3.3 The role of the PI3K/Akt pathway in COPD oxidative stress

Oxidative stress arises from an imbalance between free radicals and antioxidants, playing a significant role in inflammatory diseases (Dandekar et al., 2015). Free radicals can originate from activated inflammatory cells, structural cells, cigarette smoke, indoor and outdoor air pollution, among other sources (Valavanidis et al., 2013). COPD is a progressive respiratory disease where inflammatory and structural cells in the lungs release reactive oxygen species (ROS) and reactive nitrogen species (RNS), inducing endogenous oxidative stress during the early stages of the disease. The imbalance between free radicals and antioxidants further exacerbates ROS release (Boukhenouna et al., 2018). Thus, the elevation of oxidative stress persists even after COPD patients stop smoking. This oxidative damage results in endogenous tissue and cellular damage, ultimately leading to chronic inflammation and aging (Barnes et al., 2019). A growing body of research has demonstrated the involvement of the PI3K/Akt/mTOR signaling pathway in promoting lung cell senescence and oxidative stress (Xiaofe et al., 2022), This suggests that blocking the PI3K/Akt pathway as a means to inhibit oxidative stress could hold promise as a therapeutic strategy for COPD patients.

Recent studies have reported a significant reduction of SIRT1 and SIRT6, which are anti-aging molecules, in the lungs of COPD patients (Zhang XY. et al., 2022). Oxidative stress serves as the primary regulator of these proteins’ expression (Lakhdar et al., 2018). Inhibition of the PI3K signaling pathway, as demonstrated by Baker et al. (2016), significantly enhances the expression of SIRT1 and SIRT6 while reversing oxidative stress. Additionally, the knockout of PTEN, which inhibits PI3K signaling, resulted in reduced levels of SIRT1 and SIRT6. Studies by Xie S et al. (Xie and Wang, 2022) have shown that cigarette smoke extract downregulates the expression of CRYAB, a recognized anti-apoptotic protein, in the alveoli of COPD mice. Furthermore, overexpression of CRYAB inhibits oxidative stress, delays the activation of the PI3K/Akt signaling pathway, and reduces apoptosis. Oxidative stress also affects histone deacetylase (HDAC) activity. HDAC, a glucocorticoid functional protein, is frequently associated with glucocorticoid resistance in COPD patients (Rossios et al., 2012), Marwick et al. (2009) found reduced HDAC activity in COPD mice, and knockout of PI3K restored the activity of this enzyme. Consequently, inhibiting the PI3K pathway reinstates histone activity in the presence of oxidative stress-induced glucocorticoid resistance. This restoration, in turn, revives the anti-inflammatory properties of glucocorticoids, leading to a positive inhibition of COPD progression.

## 4 Potential drug targeting PI3K/Akt for COPD

The pathogenesis of COPD is unclear and is commonly linked to inflammation, oxidative stress and reduced immune function. Current treatments include medication, oxygen therapy and rehabilitation therapy to improve symptoms of airflow restriction caused by reduced lung function (Wang et al., 2020). However, these methods have done little to prevent the progression of COPD disease. Common COPD drugs include beta 2 receptor agonists, anticholinergic drugs, and glucocorticoids. However, chronic inhalation of beta 2 receptor agonists may have adverse cardiovascular and metabolic effects (Vanfleteren et al., 2018). There are a number of side effects associated with anticholinergic drugs, including the dry mouth, blurred vision, and postural hypotension. Glucocorticoids negatively affect the hypothalamic-pituitary-adrenal axis and most COPD patients are insensitive to glucocorticoids. Therefore, there is an urgent need to find novel molecular targeted therapeutics for COPD.

### 4.1 Application of PI3K inhibitors in COPD treatment

PI3K is a signaling cascade component downstream of multiple cell receptors. Among the three subtypes of PI3K (α, γ and δ), PI3Kα is critical for airway inflammation and angiogenesis (Chen et al., 2021), while Pl3Kγ is pro-inflammatory and involved in inflammatory cell recruitment (Heit et al., 2008) and PI3Kδ contributes to corticosteroid resistance (To et al., 2010).

Wortmannin, a PI3K inhibitor with low substrate specificity. Significantly reduced the activity of neutrophils elastase (NE) and matrix metalloproteinase-9 (MMP-9) released by airway neutrophils in COPD mice and decreased neutrophils inflammation (Vlahos et al., 2012). In addition, Wortmannin induced differentiation of alveolar epithelial stem cells in COPD mice to repair the alveoli and restore respiratory function (Horiguchi et al., 2015). LY 294002 (a non-selective PI3K inhibitor) significantly restored sensitivity to corticosteroids in PBMC cells from COPD patients, but had no effect on the production of the inflammatory factor IL-8. In addition, LY-294002 inhibits the expression of intercellular adhesion molecule −1(ICAM-1) in COPD patients, mediating monocyte/macrophage adhesion and infiltrating inflammatory sites (Liu et al., 2018).

Bewley et al. (2016) found that NVS-PI3K-2 (PI3Kα specific inhibitor), NVS-PI3K-3 (PI3Kδ specific inhibitor) and NVS-PI3K-5 (PI3Kγ specific inhibitor) suppressed lung inflammation and bacterial colonization in COPD patients. Alveolar macrophages play a significant role in clearing bacteria and small apoptotic bodies. However, these inhibitors do not alter the phagocytosis of alveolar macrophages, i.e., do not negatively affect innate immunity of COPD macrophages. Similarly, lower concentrations of theophylline (PI3Kδ specific inhibitors) can target PI3K to reverse oxidative stress-induced corticosteroid resistance and suppress lung inflammation in COPD mice exposed to cigarette smoke (To et al., 2010). In addition, IC-87114, a PI3Kδ specific inhibitor, inhibits neutrophils recruitment and restores corticosteroid sensitivity impaired under oxidative stress by inhibiting PI3K signaling (Rossios et al., 2012). Wang et al. (2019) and others demonstrated that IL-1α and IL-1β expression in human bronchial epithelial cells (HBEC) were significantly upregulated in mucin protein Muc-5ac associated with high mucin secretion, and PIK-75 (PI3Kα specific inhibitor) significantly inhibited PM-induced inflammation and mucin hypersecretion in HBEC, while AS-252244 (PI3Kγ specific inhibitor) and IC-87114 did not. Small molecule inhibitors targeting the PI3K/Akt signaling pathway treat COPD through inhibiting oxidative stress and inflammation, as shown in Figure 2.

### 4.2 Application of Akt inhibitors in COPD treatment

It is thought that Akt is a central regulator of the molecular pathways involved in smoking-related diseases, particularly COPD. MK-2206 (Akt variant non-ATP competitive inhibitor), an anticancer agent that inhibits all Akt subtypes, is commonly used to synergistically enhance the anti-tumor efficacy of certain molecular targeted drugs (Yap et al., 2011). Jiang et al. (2018) found that MK-2206 reversed changes in markers involved with epithelial mesenchymal transition (EMT) in the lung epithelium of smoking mice. EMT is positively associated with an invasive or metastatic phenotype of COPD (Zhang et al., 2016), so MK-2206 inhibits COPD. In addition, MK-2206 pretreatment inhibited IL-1α and IL-1β and Muc-5ac expression (Wang J. et al., 2019), and also protected the diaphragm in COPD mice induced by hypoxia pretreatment (Chuang et al., 2018). In addition, GSK690693 (ATP-competitive pan-Akt inhibitor) significantly inhibited IL-8 induced apoptosis in inflammatory diaphragm cells (Wang L. et al., 2019). Akt inhibitors are currently understudied and underused in COPD diseases, and more recently, Akt-negative dominant mutant (Akt-DN) transfected cells from Lin CH et al. (Lin et al., 2020) have been shown to inhibit the Akt pathway and IL-8 secretion in human lung epithelial cells.

### 4.3 Others

In addition to some of these molecular targeted drugs, natural compounds, traditional Chinese medicine formulations, and some anti-inflammatory agents also inhibit the progression of COPD by inhibiting the PI3K/Akt pathway. It has been shown recently that puerarin can relieve over-oxidation in cells (Zhang P. et al., 2022). Wang L. et al. (2022) found that puerarin reverses apoptosis in HBEC stimulated by cigarette smoke extract (CSE) via the PI3K/Akt signaling pathway. Icariin is one of the active components of Bufei Yishen formula, which can inhibit the mucus hypersecretion in COPD rats (Li J. et al., 2020). Icariin in combination with Nobilitin has a positive therapeutic effect on COPD by improving lung inflammation and emphysema and reducing lung pathological damage in COPD rats via the PI3K/Akt pathway (Lu et al., 2022). At the same time, Scutellaria has a reverse effect on lung pathologic injury induced by smoking in COPD rats (Xu et al., 2018). Crocin, the active ingredient in crocus, significantly inhibits the number of neutrophils and macrophages and the concentration of pro-inflammatory cytokines in COPD mice by modulating the PI3K/Akt-mediated inflammatory pathway (Xie et al., 2019).

As a vital component of complementary alternative medicine, TCM is considered to play its pharmacological role via its multi-component, multi-target, and multi-pathway properties (Ren et al., 2019). Notably, Bu-Shen-Fang-Fang-Chuan formula (BSFCF), a commonly used formula for treating COPD in China, attenuates the inflammatory response to COPD by inhibiting PI3K/Akt-Nrf2 and PI3K/Akt-NF-κB (Li Q. et al., 2020). Xuefu Zhuyu Decoction (XFZYD) is also widely used in the treatment of COPD, and (Hu et al., 2022) and others have found that XFZYD is effective in the treatment of COPD by interfering with the PI3K/Akt signaling pathway, improving oxidative stress and inflammatory responses, and relieving airway remodeling and ventilation disorders through web-based pharmacology and molecular docking experiments. In addition, Tiaobu Feishen (TBFS) was observed to reverse lung inflammation and airway remodeling (Li et al., 2012), Zhou et al. (2023) observed that TBFS has more effective in glucocorticoid-resistant COPD patients by modulating PI3K/Akt signaling to improve glucocorticoid resistance.

Anti-inflammatory agents that interfere with the PI3K/Akt pathway also have significant potential to improve COPD steroid resistance. Macrolides may reduce lung inflammation in COPD by modulating the PI3K/Akt pathway, such as erythromycin, which enhances corticosteroid sensitivity by inhibiting the activity of the PI3K/Akt pathway (Miao et al., 2015; Sun et al., 2015). Solithromycin (SOL, CEM-101), a macrolide/fluoronolactone that inhibits airway neutrophils in steroid-insensitive mice, is an effective anti-inflammatory agent for COPD treatment (Kobayashi et al., 2013). The statin simvastatin improves lung remodeling by reversing epithelial mesenchymal transition in alveolar epithelial cells. This effect is mediated by inhibition of the PI3K/Akt pathway (Milara et al., 2015). In addition, the tricyclic antidepressant nortriptyline can also increase corticosteroid sensitivity. Mercado et al. (2011) found that nortriptyline pretreatment inhibited Akt phosphorylation and PI3K activity, restoring oxidative stress-induced corticosteroid sensitivity as a potential treatment for respiratory diseases such as COPD that are corticosteroid insensitive.

## 5 Conclusion

The morbidity and mortality rates associated with COPD remain substantial, posing numerous challenges for healthcare professionals involved in COPD interventions. There is a growing body of evidence indicating the therapeutic potential of the PI3K/Akt signaling pathway, particularly different PI3K isoforms, in the treatment of COPD. Several non-specific PI3K inhibitors have demonstrated anti-inflammatory and antioxidant effects in COPD models. However, most broad-spectrum PI3K inhibitors exhibit greater genotoxicity, whereas PI3K subtype-specific inhibitors offer the desired therapeutic properties with reduced side effects. This may provide guidance for subsequent drug development targeting PI3K/Akt. In conclusion, there is an urgent need for further insights into the key regulatory mechanisms of the PI3K/Akt pathway in COPD development, as well as the exploration of safer and more effective therapeutic strategies derived from this approach.
